# Interactive Effects of Elevated CO_2_ and N Fertilization on Yield and Quality of Tomato Grown Under Reduced Irrigation Regimes

**DOI:** 10.3389/fpls.2018.00328

**Published:** 2018-03-27

**Authors:** Zhenhua Wei, Taisheng Du, Xiangnan Li, Liang Fang, Fulai Liu

**Affiliations:** ^1^Center for Agricultural Water Research in China, China Agricultural University, Beijing, China; ^2^Department of Plant and Environmental Sciences, Faculty of Science, University of Copenhagen, Copenhagen, Denmark; ^3^Northeast Institute of Geography and Agroecology, Chinese Academy of Sciences, Changchun, China

**Keywords:** CO_2_, nitrogen, reduced irrigation, yield, fruit quality, tomato

## Abstract

The interactive effects of CO_2_ elevation, N fertilization, and reduced irrigation regimes on fruit yield (FY) and quality in tomato (*Solanum lycopersicum* L.) were investigated in a split-root pot experiment. The plants were grown in two separate climate-controlled greenhouse cells at atmospheric [CO_2_] of 400 and 800 ppm, respectively. In each cell, the plants were fertilized at either 100 or 200 mg N kg^-1^ soil and were either irrigated to full water holding capacity [i.e., a volumetric soil water content of 18%; full irrigation (FI)], or using 70% water of FI to the whole pot [deficit irrigation (DI)] or alternately to only half of the pot [partial root-zone irrigation (PRI)]. The yield and fruit quality attributes mainly from sugars (sucrose, fructose, and glucose) and organic acids (OAs; citric acid and malic acid) to various ionic (NH_4_^+^, K^+^, Mg^2+^, Ca^2+^, NO_3_^-^, SO_4_^2-^, and PO_4_^3-^) concentrations in fruit juice were determined. The results indicated that lower N supply reduced fruit number and yield, whereas it enhanced some of the quality attributes of fruit as indicated by greater firmness and higher concentrations of sugars and OAs. Elevated [CO_2_] (*e*[CO_2_]) attenuated the negative influence of reduced irrigation (DI and PRI) on FY. Principal component analysis revealed that the reduced irrigation regimes, especially PRI, in combination with *e*[CO_2_] could synergistically improve the comprehensive quality of tomato fruits at high N supply. These findings provide useful knowledge for sustaining tomato FY and quality in a future drier and CO_2_-enriched environment.

## Introduction

As a consequence of anthropogenic activities, the carbon dioxide concentration ([CO_2_]) in atmosphere has reached 400 ppm at present, and is projected to approximately double by the end of this century (Intergovernmental Panel on Climate Change [IPCC], 2013). The elevated [CO_2_] (*e*[CO_2_]) in the atmosphere contributing to global warming causes changes in precipitation patterns and results in water scarcity in many areas, particularly in arid and semi-arid regions, where agriculture accounts for >70% of freshwater withdrawals (Wang et al., 2010). Moreover, nitrogen (N) fertilization is an essential mineral nutrient, and inadequate N supply could have significant influence on yield and quality of horticultural crops (Sainju et al., 2003), including tomato (*Solanum lycopersicum* L.), which is one of the most popular fruit crops grown around the world. Therefore, a better understanding of tomato plant responses to *e*[CO_2_], N fertilization rate, and water availability is necessary for optimizing tomato fruit yield (FY) and quality in the future climate change scenarios.

Tomato is tasty and nutritional and constitutes an important source of minerals, vitamins, and antioxidants that are essential for human health (Sun et al., 2014). The quality attributes including fruit firmness (FM), minerals, concentrations of total soluble solids (TSS), sugars, and organic acids (OAs), as well as their ratio determine not only the sweetness and sourness but also the overall flavor intensity of tomato fruits (Jones and Scott, 1984). Apart from organic components, minerals are also key quality attributes in tomatoes. Mineral elements commonly found in tomato fruit with significant amounts are N, P, K, Ca, Mg, and S, which are essential for maximizing all aspects of fruit quality (Sainju et al., 2003).

In recent years, it is of great interest to exploit appropriate reduced irrigation management strategies for sustaining crop yield and improving fruit quality in drought prone regions. Alternate partial root-zone irrigation (PRI) and deficit irrigation (DI) are effective irrigation techniques that are being investigated in many regions (Davies et al., 2002; Du et al., 2015). DI is a method that irrigates the entire root zone with an amount of water less than the potential evapotranspiration and the mild stress that develops minimal effects on the yield (Dodd, 2009). PRI is a further refinement of DI and the principle behind PRI is to alternately allow one part of the root system to be irrigated to keep the leaves hydrated while the other part is exposed to soil drying, triggering stronger root-to-shoot abscisic acid (ABA) hormonal signaling, inducing partial stomatal closure (Kang and Zhang, 2004; Liu et al., 2006), better water status in plant (Xu et al., 2009; Sun et al., 2014), greater water, and N use efficiency (Wang et al., 2010). Moreover, accumulated evidence has demonstrated that, compared with the full irrigation (FI), both DI and PRI could save up to 25–50% irrigation water without reduction in yield (Wang et al., 2010; Barrios-Masias and Jackson, 2016; Wei et al., 2016), accompanied with several significantly improved fruit quality attributes, such as firmness, TSS, and sugar to acid ratio (SAR) (Davies et al., 2000; Zegbe et al., 2003, 2004, 2006; Campos et al., 2009; Patanè and Cosentino, 2010; Wang and Frei, 2011; Hou et al., 2017), as well as ionic concentrations in fruit juice of tomatoes (Sun et al., 2014).

In addition to water management, N nutrition is also known to be an important factor affecting plant growth, yield, and quality of fruit crops (Sainju et al., 2003). A low N supply could decrease the development of the foliar canopy, trigger changes in tomato secondary metabolism, and reduce photoassimilates available into fruit, hereby influencing the firmness, TSS, sugar, and acid concentrations (Huett and Dettmann, 1988; Domis et al., 2002; Wang et al., 2007; Bénard et al., 2009).

CO_2_ plays a crucial role in physiology of plants by affecting the leaf photosynthesis, plant growth, and crop yield. Previous studies have concluded that plant photosynthesis, stomatal aperture, biomass production, yield, and water use efficiency could be modulated by CO_2_ environment (Ainsworth and Long, 2005; Sanz-Sáez et al., 2010; Pazzagli et al., 2016). For instance, more carbohydrates could be transferred into fruits due to the increased photosynthesis in plants grown under CO_2_-enriched environment, which could enhance yield and increase the concentrations of starch, sugars, ascorbic acid, and OA (Islam et al., 1996; Bindi et al., 2001; Högy and Fangmeier, 2009; Moretti et al., 2010; Sun et al., 2012). Nevertheless, there is generally a reduction in mineral contents, particularly N concentrations, in cereals grown at *e*[CO_2_] (Li et al., 2016); most probably due to restricted root nutrient uptake (i.e., caused by reduced mass flow) and dilution effect (Loladze, 2002; Myers et al., 2014). However, until now the effect of *e*[CO_2_] on mineral concentrations in fruit crops has not been examined thoroughly (Loladze, 2014), and the combined effects of *e*[CO_2_], N fertilization, and reduced irrigation regimes on fruit quality in tomato remain largely elusive.

In this study, tomato plants were grown in two atmospheric [CO_2_] (400 and 800 ppm) combined with two N fertilization rates (100 and 200 mg kg^-1^ soil) and exposed to three different irrigation regimes (FI, DI, and PRI) during flowering to fruiting stages. The yield and quality attributes including firmness and concentrations of TSS, sugars, OAs, and several minerals in fruit juice were determined. It was hypothesized that *e*[CO_2_] would ameliorate the negative effects of reduced irrigation on tomato yield; on the other hand, it would cause reduced mineral nutrition, hence decreasing the fruit quality, while PRI could improve plant nutrient uptake and increase of N fertilization might further increase plant yield and sustain fruit quality at *e*[CO_2_].

## Materials and Methods

### Experimental Setup

The experiment was conducted in a climate-controlled greenhouse at the experimental farm of the Faculty of Science, University of Copenhagen, Taastrup, Denmark, from September 2016 to January 2017. Tomato seeds (*Solanum lycopersicum* L., cv. Elin) were sown on 26th September 2016. Elin is a common indeterminate tomato cultivar being widely grown in Denmark and Sweden. The seedlings were transplanted into 1.5 L pots filled with peat substance (Sphagnum, 32% organic matter, pH = 5.6–6.4, and EC = 0.45 m^s^cm^-1^) at the fourth leaf stage. From sowing, half of the plants (24) were grown in a greenhouse cell with ambient CO_2_ concentration of 400 ppm (*a*[CO_2_]), and another half were grown in a cell with elevated CO_2_ concentration of 800 ppm (*e*[CO_2_]). In both greenhouse cells (50 m^2^ each), the [CO_2_] was sustained or achieved by emission of pure CO_2_ from a bottled tank, released in one point, and distributed evenly in the cells through internal ventilation. The [CO_2_] in the glasshouse cells was monitored every 6 s by a CO_2_ Transmitter Series GMT220 (Vaisala Group, Helsinki, Finland). The [CO_2_] concentration was kept almost constant in each cell during the whole treatment period and the data have been shown in Yan et al. (2017). The climatic conditions in the two glasshouse cells were set at: 23/16 ± 2 °C day/night air temperature, 60% relative humidity, 16 h photoperiod, and 500 μmol m^-2^s^-1^ photosynthetic active radiation supplied by sunlight plus LED lamps (Philips GreenPower LED Toplighting, Frederikskaj 6, 2450 København SV, Denmark).

Five weeks after sowing, tomato seedlings were transplanted into 10 L pots (17 cm diameter and 50 cm depth) in the greenhouse, filled with 14.5 kg of air-dried soil. All pots used were divided vertically into two equal-sized compartments with plastic sheets such that the water exchange between the two compartments was prevented. A piece of plastic (4 cm × 5 cm) was removed from the middle of the sheet where the tomato seedling was transplanted. Plant spacing was 0.4 m × 0.4 m resulting in six plants per m^2^. The soil used was classified as sandy loam, with a pH of 6.7, total C 10.3 g kg^-1^, total N 1.0 g kg^-1^, NH_4_^+^ 0.1 mg kg^-1^, and NO_3_^-^ 5.3 mg kg^-1^. The soil was sieved through 5 mm mesh before filling the pots. The soil had a volumetric soil water content (% vol.) of 18.0% and 5.0% at pot water holding capacity and permanent wilting point, respectively.

### Treatments

The experiment was conducted in two greenhouse cells, one with *a*[CO_2_] (400 ppm) and the other with *e*[CO_2_] (800 ppm). Two N (in the form of NH_4_NO_3_) fertilization rates, i.e., 100 (N1) and 200 (N2) mg kg^-1^ soil, were included and each was assigned to half of the plants in both of the greenhouse cells. The N fertilizer was mixed thoroughly with the soil before filling the pots. At the same time, 50 and 60 mg kg^-1^ soil P and K, respectively, were also applied as KH_2_PO_4_ into the soil to meet the nutrient requirements for plant growth.

The average soil water content in the pot was monitored by a time-domain reflectometer (TRASE; Soil Moisture Equipment Corporation, Santa Barbara, CA, United States) with two probes (35 cm in length) installed in the middle of each soil compartment, namely, four probes in each pot. The tomato plants were well-watered to full pot water holding capacity (i.e., a volumetric soil water content of 18%) to compensate for the evapotranspiration water loss during the previous day in the first 3 weeks after transplanting. The irrigation was done manually at 15:00 h daily. The plants were exposed to three irrigation treatments: (1) FI where both soil compartments were watered daily to a volumetric soil water content of 18%, the irrigation volume (*I*_FI_) was calculated as: *I*_FI_ = 10 × (18% - θ_mean_), where 10 is the soil volume of the whole pots and θ_mean_ is the mean soil water content of the two compartments; (2) alternative PRI, where half of the root system was watered to 70% of the FI irrigation volume while the other half was allowed to dry to ca. 6%, and then the irrigation was shifted between the two soil compartments; and (3) DI where the same amount of water for PRI was evenly irrigated to the two soil compartments. No leaching fraction was considered during the irrigation treatment.

The experiment was a complete randomized design with four replicated plants in each irrigation and N treatment at both *a*[CO_2_] and *e*[CO_2_]. Although the number of replicates for each treatment was limited, the controlled environmental conditions in the greenhouse cells were steady during experimental period, this had facilitated a similar fruit growth and development among the replicated plants for each treatment, and the difference between treatments was obvious. Thus, the data of the FY and quality should be reliable. The water used for the irrigation was tap water with negligible concentrations of nutrients. The irrigation treatments lasted 40 days and each soil compartment of the PRI plants had experienced five drying/wetting cycles.

### Measurements

The tomato variety Elin is an indeterminate type. Therefore, after the fourth fruit trusses appearance, the plants were pruned by removing the apex to stop the vegetative growth. At first truss fruits reached red maturity, namely, 64 days after plant transplanting, all the fresh fruits from the four plants of each treatment were harvested and fruit number (FN) and yield (FY) (namely, the fresh weight of all harvested fruits) per plant were recorded. Single fruit weight (SFW) of each plant was calculated as the ratio of FY to FN.

Four ripen fruits per treatment, i.e., one fresh fruit per plant in each treatment at the firm red stage, were chosen for quality measurements. FM was measured on the middle of the pericarp and reading was recorded for each fruit with a FTX Fruit Tester (Wagner Instruments, Greenwich, CT, United States) (Sun et al., 2014). Thereafter, each fruit was cut into small pieces and homogenized thoroughly in a fruit blender. The homogenate was centrifuged at 4,000 × *g* for 5 min and the supernatant was filtered through a syringe filter (0.22 mm Acetate Cameo; Osmonics, Minnetonka, MN, United States). TSS concentration of the juice was then measured using a digital refractometer with automatic temperature compensation (RFM 90; Struers Ltd., Catcliffe Rotherham, United Kingdom) and was expressed in °Brix.

Fruit juice concentrations of fructose, glucose, sucrose, citric acid, malic acid, NH_4_^+^, K^+^, Mg^2+^, Ca^2+^, NO_3_^-^, SO_4_^2-^, and PO_4_^3-^ were analyzed by ion chromatography (Metrohm AG, Herisau, Switzerland). The determination of fructose, glucose, and sucrose was done on a Metrosep Carb 1-150 column using 100 mM sodium hydroxide as eluent, while the measurement of citric and malic acid was done on a Carbohydrate H^+^ column using 0.5 mM sulfuric acid and 10% acetone as eluent. Total sugar (TS) concentration was calculated as the sum of fructose, glucose, and sucrose, and OA concentration was calculated as the sum of citric and malic acid. The SAR was also calculated. NH_4_^+^, K^+^, Mg^2+^, and Ca^2+^ concentrations were determined on a Metrosep C4-100 analytical column (4 mm × 125 mm, 1.7 mM nitric acid/0.7 mM dipicolinic acid eluent), while NO_3_^-^, SO_4_^2-^, and PO_4_^3-^ concentrations were determined on a Metrosep A Supp 4 analytical column (4 mm × 125 mm, 1.8 mM Na_2_CO_3_/1.7 mM NaHCO_3_ eluent). Total cation concentration (TCN) was calculated as the sum of NH_4_^+^, K^+^, Mg^2+^, and Ca^2+^ concentration; total anion concentration (TAN) was calculated as the sum of NO_3_^-^, SO_4_^2-^, and PO_4_^3-^ concentration; and total ionic concentration (TIN) was calculated as the sum of TCN and TAN. The above measurements were done for four samples of each treatment.

### Statistical Analyses

Three-way analysis of variance was performed for the independent variables: CO_2_ concentration ([CO_2_]), N fertilization (N), and irrigation regime (IRRI), as well as for their interactions. Data were analyzed with SPSS version 18.0 (IBM Electronics). LSD’s multiple range test was applied to assess the differences between treatments at a significance level of 5%. Principal component analysis (PCA) approach was used to evaluate the comprehensive fruit quality from FM to TIN (15 parameters in total) as affected by the different treatments. The PCA method has been detailed in Wang et al. (2015).

## Results

### Soil Water Content

**FIGURE 1:**
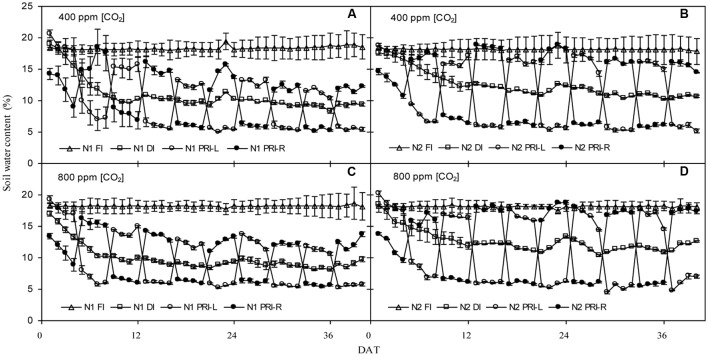
Daily average volumetric soil water content (%) in the pots of tomatoes exposed to three irrigation regimes (full irrigation, FI; deficit irrigation, DI; and alternative partial root-zone irrigation, PRI). PRI-L and PRI-R represent the left and the right soil compartment of the PRI pots, respectively, combined with two N fertilization rates (N1 and N2) under atmospheric 400 ppm [CO_2_] **(A,B)** and with two N fertilization rates (N1 and N2) under atmospheric 800 ppm [CO_2_] **(C,D)**. Error bars indicate standard error of the mean (*n* = 4).

Changes of daily average volumetric soil water content in the 0–35 cm soil profile are illustrated in **Figure 1**. Irrespective of [CO_2_] environment, the soil water contents showed similar trends for the same IRRI and N treatment. In FI, soil water contents remained at an average value of ca. 18% during the 40 days after initiation of the irrigation treatment (DAT) under both N1 and N2. In DI, soil water contents declined considerably in the first 8 DAT and remained at an average value of 10 and 12% during the last 32 days under N1 and N2 treatments, respectively. In PRI, soil water contents were dependent on which side of the root system was being irrigated. Soil water contents in the irrigated soil compartment were above 12 and 16% under N1 and N2 treatments, respectively, while soil water contents in the dry side decreased sharply and were about 6% before the shifting of irrigation under both N1 and N2 treatments.

### Fruit Number, Single Fruit Weight, and Fresh Fruit Yield

Fruit number was only significantly affected by N (**Table 1**), and was higher in plants grown under N2 than N1 treatment. SFW was not affected by any of the [CO_2_], N, and IRRI treatments (**Table 1**). Fresh FY was affected significantly by [CO_2_], N, and IRRI (**Table 1**). N2 had higher FY than N1 plants. Under N2 treatment, DI and PRI plants grown in *e*[CO_2_] had greater FY than those grown in *a*[CO_2_] environment. Across N treatments, the FI plants had similar FY to DI and PRI plants at *e*[CO_2_].

**Table 1:** Output of three-way analysis of variance (ANOVA) and mean value on measured yield and some fruit quality parameters of tomato plants as affected by the atmospheric [CO_2_] (400 and 800 ppm), N fertilization rates (N1 and N2), and irrigation regimes (full irrigation, FI; deficit irrigation, DI; and alternative partial root-zone irrigation, PRI).

Factors	FN	SFW	FY	FM	TSS	TS	OA	SAR
								
		(g)	(g plant^-1^)	(kg cm^-2^)	(°Brix)	(g L^-1^)	(g L^-1^)	
[CO_2_]	ns	ns	^∗^	ns	^∗∗^	ns	ns	^∗∗^
400 ppm	14.04	63.08	870.65	3.16	4.84	1.44	26.04	19.71
800 ppm	14.17	67.81	940.60	3.27	5.14	1.32	29.81	22.95
N	^∗∗∗^	ns	^∗∗∗^	^∗∗^	^∗∗∗^	^∗^	^∗∗∗^	^∗∗^
N1	11.17	65.38	711.81	3.38	5.27	1.61	30.08	19.75
N2	17.04	65.51	1099.44	3.06	4.71	1.15	25.77	22.92
IRRI	ns	ns	^∗∗^	ns	ns	ns	ns	^∗^
FI	14.13	70.21	977.81a	3.16	5.01	1.44	25.75	19.41b
DI	14.38	62.59	878.94b	3.21	4.88	1.39	28.43	21.29ab
PRI	13.81	63.53	860.13b	3.29	5.08	1.32	29.59	23.30a
[CO_2_] × N	ns	ns	ns	^∗^	^∗∗^	ns	^∗∗^	^∗∗∗^
[CO_2_] × IRRI	ns	ns	ns	^∗^	Ns	ns	ns	ns
N × IRRI	ns	ns	ns	ns	Ns	ns	ns	ns
[CO_2_] × N × IRRI	ns	ns	ns	^∗∗^	Ns	ns	ns	^∗∗^


*The table reported the significance results of the three-way ANOVA on total fresh fruit number (FN), single fresh fruit weight (SFW), total fresh fruit yield (FY), fresh fruit firmness (FM), total soluble solid (TSS) concentration, total sugar (TS) concentration, organic acid (OA) concentration, fruit sugar to acid ratio (SAR), and each mean value of the main factors ([CO_*2*_] 400 and 800 ppm, N1 and N2, FI, DI, and PRI) of tomato plants as affected by the CO_*2*_ environment ([CO_*2*_]), nitrogen fertilization (N), and irrigation regimes (IRRI) and their interactions.*

*^∗^, ^∗∗^, and ^∗∗∗^ indicate significance levels at *P* < 0.05, *P* < 0.01, and *P* < 0.001, respectively; ns denotes no significance.*

*Different letters at sampling data under irrigation treatments (IRRI) indicate significant differences between treatments according to LSD’s multiple range test at *P* < 0.05.*

### Fruit Firmness, Total Soluble Solids, Total Sugars, Organic Acids, and Sugar to Acid Ratio

**FIGURE 2:**
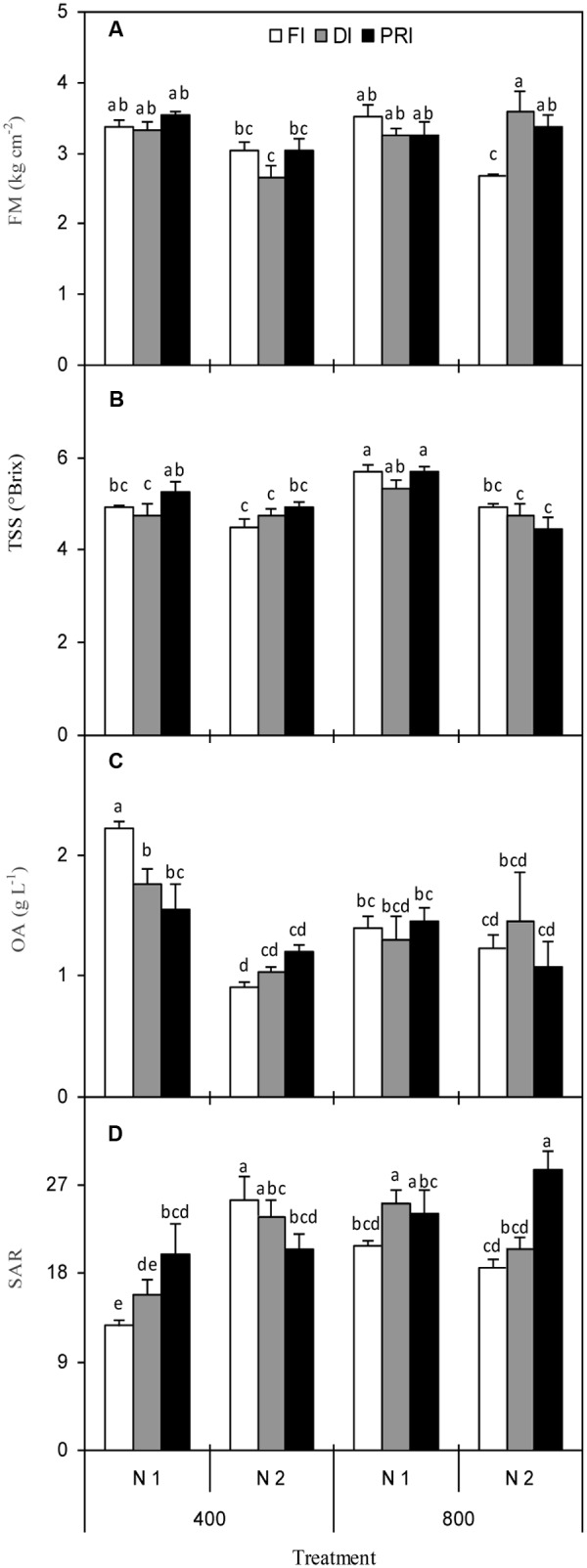
Fresh fruit firmness (FM) **(A)**, total soluble solid (TSS) concentration **(B)**, organic acid (OA) concentration **(C)**, and sugar to acid ratio (SAR) **(D)** of tomato plants as affected by the atmospheric [CO_2_] (400 and 800 ppm), N fertilization rates (N1 and N2), and irrigation regimes (FI, DI, and alternative PRI). Error bars indicate standard error of the mean (*n* = 4). Different letters at each sampling data indicate significant differences between treatments according to LSD’s multiple range test at *P* < 0.05. Statistical comparisons among the treatments are presented in **Table 1**.

Fruit firmness was significantly affected by N, [CO_2_] × N, and [CO_2_] × IRRI, as well as [CO_2_] × N × IRRI (**Figure 2A** and **Table 1**). Plants grown with reduced irrigation possessed higher FM than those exposed to FI under N2 + *e*[CO_2_] treatment. TSS concentration was affected by [CO_2_] and N as well as [CO_2_] × N (**Figure 2B** and **Table 1**), being that plants grown at *e*[CO_2_] had the same and greater TSS than those grown at *a*[CO_2_] under N1 and N2 treatment, respectively. TS was only affected by N treatment (**Table 1**), and was greater under N1 than N2. Reduced irrigation did not decrease TS as compared with FI at *e*[CO_2_]. OA was significantly affected by N and [CO_2_] × N (**Figure 2C** and **Table 1**), and the N2 plants had significantly lower OA than N1 plants. SAR was significantly affected by [CO_2_], N, and IRRI as well as [CO_2_] × N and [CO_2_] × N × IRRI (**Figure 2D** and **Table 1**). Regardless of IRRI treatments, plants grown under *e*[CO_2_] + N1 had the highest SAR than plants exposed to other treatments. Most interestingly, DI and PRI plants possessed greater SAR than FI at *e*[CO_2_] + N2 treatment.

### Cation Concentrations in Fruit Juice

**FIGURE 3:**
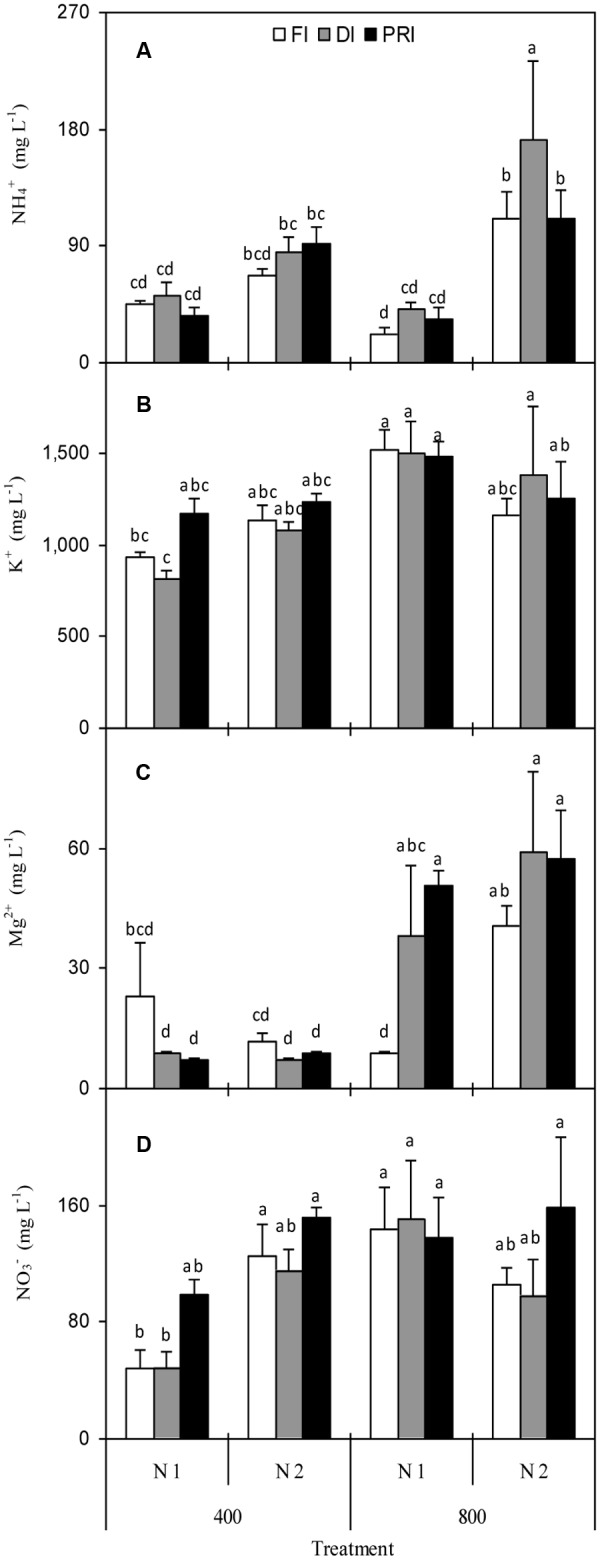
NH_4_^+^ **(A)**, K^+^ **(B)**, Mg^2+^ **(C)**, and NO_3_^-^ **(D)** concentration in fruit juice of tomato plants as affected by the atmospheric [CO_2_] (400 and 800 ppm), N fertilization rates (N1 and N2), and irrigation regimes (FI, DI, and alternative PRI). Error bars indicate standard error of the mean (*n* = 4). Different letters at each sampling data indicate significant differences between treatments according to LSD’s multiple range test at *P* < 0.05. Statistical comparisons among the treatments are presented in **Table 2**.

NH_4_^+^ concentration in fruit juice was affected by N and [CO_2_] × N (**Figure 3A** and **Table 1**). Plants grown under N2 had significantly higher NH_4_^+^ than those grown under N1 treatment, especially under *e*[CO_2_] environment. K^+^ concentration was affected by [CO_2_] and [CO_2_] × N (**Figure 3B** and **Table 2**), and *e*[CO_2_] plants had significantly greater K^+^ than *a*[CO_2_] plants. Mg^2+^ concentration was remarkably affected by [CO_2_], [CO_2_] × N, and [CO_2_] × IRRI (**Figure 3C** and **Table 2**). Disregarding N treatment, DI and PRI plants grown under *e*[CO_2_] treatment possessed higher Mg^2+^ than those grown under *a*[CO_2_] treatment. Ca^2+^ concentration in fruit juice was only significantly affected by N treatment (**Table 2**), and was greater for N2 than N1 plants.

**Table 2:** Output of three-way ANOVA and mean value on fruit quality parameters of overall ion concentration in tomato plants as affected by the atmospheric [CO_2_] (400 and 800 ppm), N fertilization rates (N1 and N2), and irrigation regimes (FI, DI, and alternative PRI).

Factors	NH_4_^+^	K^+^	Mg^2+^	Ca^2+^	NO_3_^-^	SO_4_^2-^	PO_4_^3-^	TCN	TAN	TIN
										
						(mg L^-1^)				
[CO_2_]	ns	^∗∗^	^∗∗∗^	ns	^∗^	ns	ns	^∗∗∗^	ns	^∗∗^
400 ppm	63.05	1062.93	11.09	24.15	97.85	181.84	614.71	894.40	1161.21	2055.61
800 ppm	82.15	1387.05	42.40	24.21	132.17	217.91	643.03	993.11	1535.80	2528.91
N	^∗∗∗^	ns	ns	^∗^	ns	ns	ns	ns	ns	ns
N1	38.57	1238.95	22.77	22.27	104.51	201.67	603.25	909.43	1322.56	2231.99
N2	106.63	1211.03	30.72	26.09	125.51	198.08	654.48	978.07	1374.46	2352.53
IRRI	ns	ns	ns	ns	ns	ns	ns	ns	ns	ns
FI	61.68	1190.31	21.02	22.12	105.63	193.52	611.98	911.13	1295.14	2206.26
DI	87.84	1196.74	28.22	24.09	102.72	201.02	636.53	940.27	1336.89	2277.15
PRI	68.28	1287.91	30.99	26.33	136.68	205.10	638.09	979.87	1413.51	2393.37
[CO_2_] × N	^∗^	^∗^	^∗^	ns	^∗∗^	ns	ns	ns	ns	ns
[CO_2_] × IRRI	ns	ns	^∗^	ns	ns	ns	ns	ns	ns	ns
N × IRRI	ns	ns	ns	ns	ns	ns	ns	ns	ns	ns
[CO_2_] × N × IRRI	ns	ns	ns	ns	ns	ns	ns	ns	ns	ns


*The table reported the significance results of the three-way ANOVA on cation concentration in fruit juice (NH_*4*_^+^, Na^+^, K^+^, Mg^*2*+^, and Ca^*2*+^), anion concentration in fruit juice (NO_*3*_^-^, SO_*4*_^*2*-^, and PO_*4*_^*3*-^), total cation concentration (TCN) in fruit juice, total anion concentration (TAN) in fruit juice, total ionic concentration (TIN) in fruit juice, and each mean value of the main factors ([CO_*2*_] 400 and 800 ppm, N1 and N2, FI, DI, and PRI) of tomato plants as affected by the CO_*2*_ environment ([CO_*2*_]), nitrogen fertilization (N), and irrigation regimes (IRRI) and their interactions.*

*^∗^, ^∗∗^, and ^∗∗∗^ indicate significance levels at *P* < 0.05, *P* < 0.01, and *P* < 0.001, respectively; ns denotes no significance.*

### Anion Concentrations in Fruit Juice

NO_3_^-^ concentration in fruit juice was affected by [CO_2_] and [CO_2_] × N (**Figure 3D** and **Table 2**). NO_3_^-^ for *a*[CO_2_] plants was significantly lower than those for *e*[CO_2_] plants. SO_4_^2-^ and PO_4_^3-^ concentrations, however, were unaffected by any of the [CO_2_], N, and IRRI treatments (**Table 2**).

### Total Cation, Anion, and Ionic Concentrations in Fruit Juice

Total cation concentration in fruit juice was only significantly affected by [CO_2_] treatment (**Table 2**); *e*[CO_2_] plants had significantly greater TCN than *a*[CO_2_] plants. TAN was unaffected by any of the [CO_2_], N, and IRRI treatments (**Table 2**). TIN was only remarkably affected by [CO_2_] treatment (**Table 2**), and was greater in plants grown at *e*[CO_2_] than those grown under *a*[CO_2_] environment.

### PCA Evaluation of Comprehensive Fruit Quality

**FIGURE 4:**
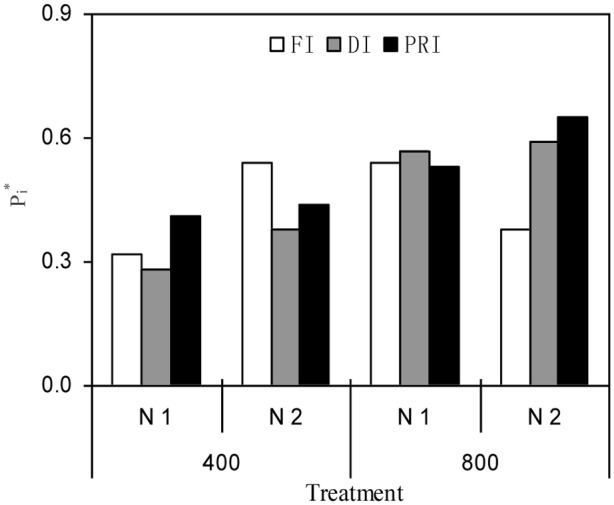
PCA evaluation of comprehensive tomato quality attributes (15 parameters) among all treatments (the atmospheric [CO_2_] (400 and 800 ppm), N fertilization rates (N1 and N2), and irrigation regimes (FI, DI, and alternative PRI). *P*_i_^∗^ value indicates the closeness between principal components in each treatment.

The PCA revealed that PRI and DI plants grown under N2 and *e*[CO_2_] environment had the highest *P*_i_^∗^ (0.65 and 0.59) values, leading to the first and second rank of comprehensive fruit quality, respectively, among all treatments (**Figure 4**).

## Discussion

It is well known that a low N supply could reduce the vegetative growth, number of fruit sets, and yield of fruits (Huett and Dettmann, 1988). In good agreement with this, here, N1 plants had significantly lower FN and FY than N2 plants, regardless of [CO_2_] and IRRI treatments (**Table 1**). Nonetheless, SFW was similar among treatments (**Table 1**). Thus, the reduced FY for lower N fertilization was attributed mainly to the decreased FN. It has been suggested that plants grown in limited N possess lower photosynthetic capacity, and reduced photoassimilates from source leaves to be translocated into the fruits, hereby decreasing fruit set and FY (Bénard et al., 2009). Likewise, plants grown under *e*[CO_2_] possessed greater FY than those grown under *a*[CO_2_] treatment. This is consistent with the findings by Domis et al. (2002); Ainsworth and Long (2005), and Pazzagli et al. (2016) that *e*[CO_2_] environment could enhance the yield or biomass production of crop. The reduction in oxygenase activity of Rubisco at *e*[CO_2_] plants could lead to a decrease of photorespiration rate and in turn to an increase on net photosynthesis (Ainsworth and Long, 2005). Therefore, sustaining higher rates of photosynthesis by either sufficient N supply and/or in combination with *e*[CO_2_] could substantially enhance FY in tomato plant (Moretti et al., 2010). On the other hand, for the same N and irrigation treatment, there was no difference in soil water content between the *e*[CO_2_] and *a*[CO_2_] treatments during the treatment period (**Figure 1**), indicating that *e*[CO_2_] plants consumed similar amount of water compared with *a*[CO_2_] plants (Wei et al., 2018). Thus, an improvement of water use efficiency for fruit production could be expected (data not shown).

Previous studies have frequently observed that both DI and PRI could consume 25–50% less water without significant reduction in FY or biomass compared to FI (Kang and Zhang, 2004; Zegbe et al., 2004, 2006; Wang et al., 2010; Du et al., 2015; Barrios-Masias and Jackson, 2016; Wei et al., 2016), although several reports revealed a significant reduction of tomato FY in PRI compared with FI (Kirda et al., 2004; Zegbe et al., 2004). In addition, studies have shown that PRI was superior to DI in improving FY (Dodd, 2009) and fruit quality (Sun et al., 2013, 2014) in tomatoes. In the present study, it is notable that the FY of plant grown under reduced irrigation (DI and PRI) was largely sustained when compared to FI treatment at *e*[CO_2_], whereas this response was not seen in *a*[CO_2_] environment where significant reduction of FY due to reduced irrigation was observed (**Table 1**). Hereby, this result clearly demonstrated that plants grown in the *e*[CO_2_] environment could attenuate the negative influence of water stress on yield, which was in good agreement with earlier findings (Zhou et al., 2014; Mamatha et al., 2015; Pazzagli et al., 2016).

This study found that DI and PRI plants possessed greater FM than FI with N2 under *e*[CO_2_] environment (**Figure 2A** and **Table 1**), implying that better tomato fruit transport and storage quality was achieved when exposed to reduced irrigation associated with *e*[CO_2_] treatment. Also, plants grown at N1 generally possessed greater concentrations of TSS, TS, and OA than those grown at N2 (**Figures 2B,C** and **Table 1**). As it is well known, N level has a strong effect on the amounts of primary and secondary metabolites in fruits (Bénard et al., 2009). Simonne et al. (2007) reported that increased OA content was associated with decreased N supply; however, other researchers found the opposite results (Huett and Dettmann, 1988; Wang et al., 2007). Such discrepancy could be due to the different cultivars and experimental conditions as well as the source–sink balance in the plants.

The concentrations of TS, OA, and SAR are the primary biochemical fruit quality attributes that contribute to the flavor in tomatoes (Domis et al., 2002). Here, under N1 fertilization, the *e*[CO_2_] plants had higher TSS and TS and lower OA than *a*[CO_2_] plants (**Figures 2B,C** and **Table 1**). Also, reduced irrigation, especially PRI plants tended to increase TS, decrease OA, and contributed to significantly greater SAR in relation to the FI under N1 + *a*[CO_2_] and N2 + *e*[CO_2_] treatments (**Figures 2C,D** and **Table 1**). It is widely accepted that reduced irrigation could maintain or enhance various aspects of tomato fruit quality (Zegbe et al., 2003; Campos et al., 2009; Xu et al., 2009; Patanè and Cosentino, 2010; Sun et al., 2014). The higher TS and lower OA together with higher SAR in fruit juice under reduced irrigation regimes indicate an improvement of fruit sweetness (Wang and Frei, 2011; Hou et al., 2017). As for the increased TSS and TS concentrations in tomato fruit, it is probably ascribed to the modulation of underlying physiological mechanisms. First, the reduction of side shoots and reproductive growth in reduced irrigation-treated plants result in a relative improvement of the sink activity in tomato fruit; thus, the carbohydrate previously partitioned toward the side shoots is redirected toward into the fruit, accompanied by the increased availability of assimilates for the remaining fruits (Davies et al., 2000; Patanè and Cosentino, 2010). Second, moderate water stress could induce a greater starch accumulation in the fruits during the earlier stage of fruit development, along with the higher conversion of starch to sugars during fruit maturation (Zegbe et al., 2003). Third, the activities of carbohydrate-metabolizing enzymes, primarily invertase plays an important role in modulating sugar concentration in tomato fruits and its activity could be modulated by ABA (Ruan et al., 2010; Sun et al., 2014). Reduced irrigation, especially PRI had the ability to induce higher ABA concentration in the tomato plants (Wang et al., 2010; Sun et al., 2013). Thereby, this hormone may stimulate invertase activity and trigger expected greater sugars in the fruit (Ruan et al., 2010).

The beneficial effects of CO_2_ enrichment on photosynthetic rate and fruit quality of crops including tomato have been extensively reported by numerous studies (Islam et al., 1996; Bindi et al., 2001; Högy and Fangmeier, 2009; Moretti et al., 2010; Sun et al., 2012). Increases in rubisco content and activity result in enhanced leaf photosynthesis, which probably lead to accumulated concentration of carbon-based compounds in response to *e*[CO_2_] environment due to the source–sink balance hypothesis (Peñuelas and Estiarte, 1998), such as TSS, diverse sugars, and acids. Furthermore, in this study, there was no decrease in water consumption at *e*[CO_2_], which could facilitate the transport of assimilates into fruits and anticipate to induce greater increase in the flavor components of fruits.

The quantity and ingredient of minerals mainly macronutrient in the fruit have a prominent effect on the nutritional quality of tomatoes (Domis et al., 2002). Here, it was found that high N supply resulted in greater NH_4_^+^ and NO_3_^-^ concentrations in fruit juice under PRI (**Figures 3A,D** and **Table 2**), confirming the fact that N deficiency could lead to the inadequate N uptake in the plant. Moreover, regardless of N fertilization, *e*[CO_2_] plants combined with reduced irrigation commonly showed no decrease in concentrations of NH_4_^+^, Ca^2+^, SO_4_^2-^, PO_4_^3-^ and total anion (TAN) or increase in concentrations of K^+^, Mg^2+^, NO_3_^-^, total cation (TCN) and total ion (TIN) in fruit juice (**Figure 3** and **Table 2**). It is well demonstrated that soil water dynamics under reduced irrigation, especially PRI induced drying and wetting cycles in the soil can have a predominantly positive influence on the bioavailability of mineral nutrients and their movement from the bulk soil to the roots of plant (Wang et al., 2010), and may enhance the root acquisition of mineral elements thereby increasing the nutritional status of the plants (Wang et al., 2012), including the ionic concentrations in the tomato fruits (Sun et al., 2013, 2014). Furthermore, due to an enhanced xylem connection to the fruits, the proportion of the ions in the plants allocated to the fruit could also be enhanced by the reduced irrigation treatments, particularly PRI (Davies et al., 2000).

It has been reported that across diverse tissues and experimental conditions, there is a nearly 8% reduction in the overall mineral content of plants grown under *e*[CO_2_] environment (Loladze, 2014), which is mainly attributed to the decreased mass flux for nutrients transport from bulk soil to root surface, limited root nutrient acquisition capacity, and dilution by a greater plant biomass (Loladze, 2002; Myers et al., 2014). In the present study, the *e*[CO_2_] plants showed similar water consumption with the *a*[CO_2_] plants, implying the equivalent mass flow at *e*[CO_2_] compared to *a*[CO_2_]. Additionally, studies have indicated that the proportion of assimilates allocated to roots is enhanced at *e*[CO_2_], resulting in a larger root system which could enhance the ability to absorb more minerals from the surrounding soil and improve the ionic contents in fruits (Idso and Idso, 2001; Wullschleger et al., 2002). Therefore, the expected negative impacts of drought stress and *e*[CO_2_] on tomato ions uptake were not evident in this study, and concentration of some minerals in fruit juice could be enhanced under *e*[CO_2_] combined with PRI strategy.

It is well known that PCA approach is a popular tool for statistical analysis and plays an important role in featuring extraction and dimensionality reduction, further realizing the accurate integrated appraisement on the variance of data source (Wang et al., 2015). In this study, the PCA assessment of comprehensive fruit quality attributes including 15 different parameters indicated that plants grown under sufficient N supply together with *e*[CO_2_] condition, the comprehensive fruit quality of deficit irrigation-treated plants, particularly for PRI, was superior to those under other treatments (**Figure 4**). Therefore, a greater fruit quality could be achieved under PRI associated with *e*[CO_2_] environment.

## Conclusion

Conclusively, low N supply decreased tomato FN and yield but increased fruit quality across irrigation and [CO_2_] treatments. Plants grown in *e*[CO_2_] environment could eliminate the negative impact of reduced irrigation on FY. Moreover, at adequate N fertilization rate, an improvement of comprehensive fruit quality could be achieved by growing in *e*[CO_2_] environment combined with reduced irrigation regimes, particularly with the PRI strategy. These findings are of great relevance for formulating agronomical practices to sustain tomato yield and quality in a future drier and CO_2_-enriched environment.

## Author Contributions

ZW: done the experiments and finished the first manuscript. TD and FL: supervised the work. XL and LF: helped to do the experiments.

## Conflict of Interest Statement

The authors declare that the research was conducted in the absence of any commercial or financial relationships that could be construed as a potential conflict of interest.
